# Developments in public health paramedicine: exploring the professional practice of ambulance clinicians in palliative and end-of-life care in a remote and rural setting

**DOI:** 10.29045/14784726.2025.9.10.2.49

**Published:** 2025-09-01

**Authors:** Lisa Kamphausen, Els Freshwater

**Affiliations:** Robert Gordon University ORCID iD: https://orcid.org/0000-0002-5295-5066; Robert Gordon University ORCID iD: https://orcid.org/0000-0003-2805-7044

**Keywords:** end-of-life care, paramedic, public health

## Abstract

**Aims:**

Professional practice in paramedicine is evolving rapidly, and with this evolution comes a growing ability – and responsibility – for paramedics to contribute to public health. Palliative and end-of-life care (PEOLC) public health is one such area where paramedicine has begun to contribute substantially and might still have significant untapped potential.

This article explores developments in PEOLC paramedicine in the Scottish Highlands, an area classified as remote and rural, characterised by low population density, widely spaced communities and susceptibility to health inequalities created by access to healthcare, especially to specialist services. The role of paramedicine in PEOLC is examined in the context of public health priorities and policy, while considering the ability of paramedics to reduce health inequalities by widening access.

**Background:**

An informal literature search was conducted to identify interventions through which paramedicine can make improvements to the experience of death and dying on a population level, and lead to substantial healthcare cost savings. These interventions range from reducing PEOLC hospital admissions through effective use of advance care planning, just-in-case medications and independent prescribing and local referral pathways, to effectively managing palliative emergencies amenable to treatment in hospital.

**Conclusion:**

Paramedicine could thus play a significant role in making policy ambitions in PEOLC a reality, and conversely, achieving PEOLC policy ambitions might be difficult without support from paramedicine. Paramedics play a growing role in community healthcare provision, especially in remote and rural settings, by providing a link between care provided in the community and specialist services. Better integration of paramedicine into primary and secondary healthcare systems could facilitate turning more PEOLC public health theory into practice. The information collated in this discussion reinforces the need to reflect this potential in research funding allocation, in social and government policy development and in clinical practice decisions made by each individual paramedic.

## Introduction

The role of paramedicine in Scotland has evolved markedly from the transportation-focused origins of Aneurin Bevan’s time to the much broader scope of practice today, in which paramedics work autonomously in a wide range of settings encompassing, for example, ambulances, emergency rooms and general practice (College of Paramedics, 2021; Eastwood et al., 2023). With this evolution has come a growing ability – and responsibility – for paramedics to contribute to public health. The rapid advances underway in public health paramedicine have created an opportunity to use the latest research to develop new policy and implement current evidence-based practice. Palliative and end-of-life care (PEOLC) is an area of public health where paramedicine is beginning to contribute, and might still have significant untapped potential (Blackmore, 2022). In the context of this article, palliative care refers to non-curative care provided to people with life-shortening conditions aiming to maximise quality of life, while end-of-life care refers to care provided when people have entered the active dying stage and death is expected within days.

This article explores developments in PEOLC paramedicine using the example of the Scottish Ambulance Service (SAS) and the population of the Scottish Highlands. Drawing on the latest research in public health and PEOLC, health informatics and health economics, the role of paramedicine in PEOLC is examined in the context of public health priorities and policy, while considering the ability of ambulance clinicians to influence the drivers of health inequalities.

Several interventions are highlighted through which paramedicine is beginning to have a substantial impact on the experience of death and dying on a population level.

## PEOLC, public health and paramedicine

Both PEOLC and paramedicine are important elements of public health.

In 2014 the World Health Organization (WHO) passed a resolution making the provision of palliative care via national health policies a global requirement (WHO, 2021). Leading up to this, PEOLC began to be recognised as an integral part of public health around the time of WHO’s Ottawa Charter (WHO, 1986), which formalised a ‘new’ public health approach with a shift away from traditional disease control and towards health promotion and community empowerment, with the key principles of advocate, mediate and enable.

Around the same time, a palliative public health approach promoting ‘compassionate communities’ emerged, which corresponded with the principle that a healthy society is the responsibility of everybody rather than that of the health service alone (Kellehear, 2013). Compassionate communities engage a whole community in addressing a person’s holistic needs in their final stages of life, in contrast to having a health system overtreat medical needs while not meeting many others (Kellehear, 2013; Sallnow et al., 2016, 2022).

The Scottish Government responded to the 2014 WHO resolution with a commitment that access to palliative care was to be available to everybody in Scotland by 2021, captured in the Strategic Framework for Action for PEOLC (Scottish Government, 2015). PEOLC policy in Scotland has been shaped by the Ottawa principles, with advocacy having resulted in a recent revision of the Scottish Palliative Care Guidelines (Health Improvement Scotland, 2019). Mediation works on reconciling the interests of individuals when nearing death with the interests of wider society and the requirement to operate within available budgets. Efforts to empower people and communities are evident in increasing use of advance care planning, enabling people to promote their own health by recording their wishes (Thomas & Russell, 2023).

Ongoing delivery of this government policy is led by the NHS, but relies heavily on social policy. In the Scottish Highlands, for example, a geographical area the size of Belgium dominated by remote and rural communities, there is only one hospice, which is an independent charity. The Highland Hospice receives a grant from the NHS but raises 76% of its income from other sources (Highland Hospice, 2023a). It is estimated that 90% of PEOLC in the Scottish Highlands is provided by family, carers and community members (Highland Hospice, 2023b). These figures do suggest successful community empowerment policies. A downside of this mechanism of PEOLC provision, however, is that it can leave people who do not have social support without care, relying only on crisis response by the ambulance service, which highlights the importance of paramedicine as a safety net in PEOLC (Richards et al., 2024). Paramedicine also provides a link between communities and the medical system, able to intervene in complex cases where more advanced medical input is required – either by providing this in the community or by being able to refer or convey to alternative destinations (Ahmed et al., 2023; Juhrmann et al., 2023). Paramedics can thus support both public health and PEOLC. Paramedic contributions to health promotion in PEOLC should continue to apply these guiding principles, and the most recent UK paramedic pre-registration curriculum’s emphasis on public health responsibilities of paramedics reflects UK policy supporting this development (College of Paramedics, 2024).

Another central objective of public health is the reduction of health inequalities, or unfair and avoidable differences in health outcomes in a population (Finch et al., 2023). Varying availability of healthcare services to people contributes to health inequality, which is also driven by determinants of health, such as income, education, ethnicity, sex and age (Care Quality Commission, 2016; Finch et al., 2023). Vulnerabilities generated by these determinants often affect people across multiple domains, producing multiple deprivations (Scottish Government, 2020). Inequality due to access is a greater problem in rural and remote regions, such as the Scottish Highlands, than in population centres (Scottish Government, 2020). Since paramedicine, via ambulance services, is a form of mobile healthcare that is available to everybody at any time, paramedics can facilitate more equitable access to PEOLC (Juhrmann et al., 2023). Paramedics have contact with populations who do not routinely engage with other services, and they can then initiate palliative or end-of-life care to optimise people’s quality of life and prevent complications requiring hospital admission (Juhrmann et al., 2023).

A recent study that investigated the experience of dying at home for people living with deprivation in Scotland reported that people living in areas with higher deprivation had less access to social support (Richards et al., 2024). As PEOLC provision depends highly on social policy, the backstop of its provision by ambulance clinicians may be especially important in areas of higher deprivation. Deprivation in Scotland is recorded through the Scottish Index of Multiple Deprivation (SMID), according to which only 10% of data zones in the Highlands were part of the most deprived quintile in 2020 (Scottish Government, 2020). While this might suggest that deprivation may not affect PEOLC provision for many people in the Highlands, there are limitations to the inferences that can be drawn from SIMD data in sparsely populated areas. The heterogeneity within data zones in rural areas is greater than in densely populated zones, so there are people in deprivation in Highlands who are not represented by SIMD data because they live in small pockets of overall less-deprived data zones. Additionally, a central component of deprivation in rural and remote areas is inability to access services because of travel distances (Scottish Government, 2016b). The Highland Hospice Palliative Care Response Service, for example, which can provide short-notice home care for people in their final weeks of life, is available in Inverness only (Highland Hospice, 2023a), which results in outlying areas having to make greater use of, for example, ambulance services.

By widening access to PEOLC, paramedicine is thus able to help reduce the gaps between policy ambitions and practice, and to help address the conflict that can occur between the goals of reducing inequalities and of empowering communities in favour of centralised provision of services, which can leave marginalised people falling through the gaps (Juhrmann et al., 2023; Pekarsky et al., 2021) .

## Health economics and paramedic PEOLC

The context within which PEOLC policy has developed is that of death and dying as taboo subjects in the western world. The understanding of dying as a failure of medicine, rather than a natural and expected process, has created a general helplessness in the face of death, while enormous efforts are directed towards fighting it, including use of interventions with very small chances of success or which ultimately prolong suffering (Illich, 1976; Mannix, 2017; Sallnow et al., 2022). Gawande (2015) likens this approach to issuing people medical lottery tickets without preparing them for the very high likelihood that their tickets will not win. Consequently, western healthcare systems deploy vast resources during the last year of a person’s life, leading to spiralling costs of treatment of an ageing population (Diernberger et al., 2021). PEOLC has become recognised as a possible solution to this problem, by optimising quality of life and mitigating physical, psychosocial and spiritual suffering towards the end of life, and almost coincidentally also producing substantial reductions in healthcare costs (Collins et al., 2021; Diernberger et al., 2021; Krakauer et al., 2009; Sepulveda et al., 2002; Spettell et al., 2009).

PEOLC interventions are unusual in public health in that they do not rely predominantly on influencing upstream causes of morbidity and mortality but can have significant benefits even when implemented reactively (McKinnon, 2021). PEOLC public health measures are not aiming to prevent death but to improve the experience of death and dying. The ability of PEOLC to both create better health outcomes and make treatment less expensive has been reviewed by Gawande (2015), among others. For example, in 2004, to curb spiralling end-of-life treatment costs, an American insurance company permitted policy holders with prognoses of under a year to access palliative care concurrently with, rather than instead of, curative care. Overall healthcare costs for patients who received PEOLC in addition to curative treatment fell by 25% and hospital admissions were reduced by 66%. Further studies found that people who used PEOLC had a better quality of life and remarkably lived up to 25% longer than those who pursued only ‘curative’ treatment. In addition, a blanket roll-out of advance care planning, specifying a ceiling of care, reduced healthcare costs of a person’s final six months to half the national average (Gawande, 2015).

While these findings from the United States may not be directly transferable to the socialised healthcare system in the United Kingdom, a 2021 study on the healthcare costs of all Scottish decedents over the age of 60 showed consistency with US findings: secondary care costs rose exponentially during the final year of life to, on average, £10,000 per person (Diernberger et al., 2021). In the Highlands, 2200 people died in 2022 (Highland Hospice, 2023b), making this a significant expenditure. A limitation of the Scottish study is that it did not also consider primary care use, which likely makes the paper’s figure an underestimate. The greatest cost was created by inpatient hospital stays in a person’s final three months of life (Diernberger et al., 2021), even though most people wished not to die in hospital (Finucane et al., 2019). Therefore, some end-of-life hospital admissions were likely neither medically beneficial nor in keeping with the person’s wishes, while also going against the national ‘Realistic Medicine’ policy, which introduced the expectation that the health system will minimise interventions that do not add value to patient care, and which would therefore contravene one of the core principles of a socialised healthcare system of trying to achieve the most for the most while operating within the constraints of limited national resources (Scottish Government, 2016a).

Ambulance clinicians, who often attend end-of-life-emergencies or when a person enters the dying phase, are instrumental in reducing unhelpful hospital transfers or futile resuscitation attempts (Collier et al., 2023; Gage et al., 2023). While the drivers behind reducing deaths in hospital are complex, progress is evident as the proportion of deaths occurring in hospital in Scotland has decreased from 58% to 50.1% between 2004 and 2014, and is projected to drop to around 33% by 2040 provided community social care provision is increased sufficiently to support this drop (Finucane et al., 2019). Considering the UK’s aging population and persistent pressures on health budgets, PEOLC as an effective route to healthcare cost savings seems an obvious area for further development in paramedicine.

Much of the existing research on the effectiveness of public health interventions in PEOLC concerns advance care planning (Scherrens et al., 2018). Advance care planning is useful, however, only if the information is accessible when required (Finucane et al., 2020). Ambulance clinicians face the challenge of encountering patients unknown to them in the community, while needing to make immediate decisions on appropriate interventions. Health informatics solutions, such as the Key Information Summary (KIS) now available to SAS clinicians via electronic care co-ordination systems, have made it possible for ambulance clinicians to access elements of advance care plans such as resuscitation status and preferred place of care and death (Birtwistle et al., 2022; Finucane et al., 2020). A study on the effectiveness of KISs in Scotland found that the proportion of people who died in the community versus in hospital was twice as high for people with a KIS than for those without (60% versus 30%) (Finucane et al., 2020). While this difference could indicate that availability of a KIS halves the number of end-of-life hospital admissions, it could also merely show that people who lack the resources to arrange dying outside hospital are also less likely to have a KIS. Thus, KISs might not facilitate community deaths as much as be correlated with people who can and want to die in the community. Nevertheless, KISs were considered useful by out-of-hours care providers (Finucane et al., 2020), and technology making information on resuscitation status, ceiling of care and preferred place of death accessible in the community is clearly invaluable to ambulance clinicians encountering patients in PEOLC emergencies.

Following this assessment of the potential of paramedicine to contribute to public health improvement and healthcare cost savings by implementing PEOLC interventions, the following section highlights some promising interventions.

Ambulance clinicians can support symptom relief in death and dying in the community by administering just-in-case medications, which are prescribed to palliative patients in advance, to use in the event of palliative emergencies. While these medications are ordinarily outside the scope of ambulance clinicians, once prescribed they can be administered by attending crews. In addition, Advanced Paramedics can be independent prescribers themselves if appropriately trained and governed, and are then able to prescribe these medications on scene when required. Hospital transfers can therefore be avoided, for example by treating respiratory distress or agitation sufficiently to enable the process of dying to proceed at home without causing undue distress to the dying person or those close to them (Blackmore, 2022).

It is, however, equally important that paramedics recognise any palliative emergencies that do require time-critical hospital intervention. As generalist clinicians who assess the whole spectrum of potentially very sick patients, paramedics routinely make complex decisions to distinguish ‘red flag’ differential diagnoses that must not be missed and require time-critical intervention from lower acuity presentations, and are therefore ideally placed to recognise palliative emergencies. Conditions such as hypercalcaemia, superior vena cava syndrome or metastatic spinal cord compression (MSCC) can significantly lower quality of life, while treatment carries a low burden and is usually successful. A timely identification of MSCC with urgent transfer to treatment may prevent development of paraplegia and incontinence, for example (Blackmore, 2022). Programmes to increase paramedic proficiency in PEOLC include the SAS and Palliative Care ECHO network (Marie Curie, 2024) and the Macmillan and SAS PEOLC project, which recently produced a widely distributed poster to reduce uncertainty around Do Not Attempt Cardio-Pulmonary Resuscitation (DNACPR) forms (Scottish Partnership for Palliative Care, 2023).

As well as providing an immediate crisis response, paramedics can use patient encounters to encourage advance care planning, and to signpost to support services. In the Highlands, paramedics can signpost to the Palliative Care Helpline, which is a single-point-of-access-service delivered jointly by the NHS and Highland Hospice, providing holistic advice and support and a wide range of referrals (Highland Hospice, 2023a).

## Conclusion

This examination of the evolving relationship between paramedicine, PEOLC and public health considered the potential for paramedicine to link community provision of care with medical interventions where required, and to reduce health inequalities by improving access, using the example of SAS and the population of the Scottish Highlands. The Highlands is a region of Scotland characterised by its low population density and widely spaced communities and is susceptible to health inequalities created by access to healthcare, especially to specialist services. Equally, in remote areas, long distances between hospitals and communities heighten the importance of enabling people to remain in their communities, to avoid isolating them in a location where few of their non-medical needs will be met. Paramedics providing PEOLC, who are able to access all areas at all times, can give people the confidence to remain in their communities, knowing that in emergencies more specialist support is available. Lessons learned here are applicable to remote and rural areas elsewhere, both in the UK and internationally.

Interventions were highlighted through which paramedicine can make improvements to the experience of death and dying on a population level, and lead to substantial healthcare cost savings.

Implications of the broad range of existing and proposed interventions are that paramedicine could play a significant role in making policy ambitions in PEOLC a reality, and conversely that achieving PEOLC policy ambitions might be difficult without support from paramedicine.

Interdisciplinary working of all levels of health and care professionals, recognition of the growing role ambulance clinicians play in community healthcare provision and better integration of paramedicine into primary and secondary healthcare systems could facilitate turning more PEOLC public health theory into practice.

The information collated in this discussion reinforces the importance of PEOLC and paramedicine to public health, and the need to reflect this importance in research funding allocation, in social and government policy development and in clinical practice decisions made by each individual paramedic.

## Author contributions

LK is the lead author of this manuscript. EF contributed to concept development, manuscript preparation, editing and submission. Both authors have reviewed and approved the final version of the manuscript. LK acts as the guarantor for this article.

## Conflict of interest

None declared.

## Ethics

Not required.

## Funding

None.

## Statement of generative AI in scientific writing

The authors did not use a generative artificial intelligence (AI) tool or service to assist with preparation or editing of this work.
